# Hearing impairment and dementia: cause, catalyst or consequence?

**DOI:** 10.1007/s00415-025-13140-x

**Published:** 2025-05-16

**Authors:** Benjamin A. Levett, Avinash Chandra, Jessica Jiang, Nehzat Koohi, Dale Sharrad, Lucy B. Core, Jeremy C. S. Johnson, Madison Tutton, Tim Green, Dona M. P. Jayakody, Jin-Tai Yu, Iracema Leroi, Charles R. Marshall, Doris-Eva Bamiou, Chris J. D. Hardy, Jason D. Warren

**Affiliations:** 1 Dementia Research Centre, UCL Institute of Neurology, Queen Square, UCL, 8-11 Queen Square, London, WC1N 3AR UK; 2 Centre for Preventive Neurology, Wolfson Institute of Population Health, Queen Mary University of London, Charterhouse Square, London, EC1M 6BQ UK; 3 Department of Clinical and Movement Neurosciences, Institute of Neurology, UCL, 33 Queen Square, London, WC1N 3BG UK; 4 The Ear Institute, UCL, 332 Grays Inn Rd, London, WC1X 8EE UK; 5 National Hospital for Neurology and Neurosurgery, Queen Square, London, WC1N 3BG UK; 6 Basic and Clinical Neuroscience, King’s College London, 5 Cutcombe Road, London, SE5 9RT UK; 7 Eargym Ltd, 63-66 Hatton Garden, London, EC1N 8LE UK; 8 Speech, Hearing and Phonetic Sciences, UCL, Chandler House, 2 Wakefield Street, London, WC1N 1PF UK; 9 University of Western Australia, 35 Stirling Hwy, Crawley, WA 6009 Australia; 10 Huashan Hospital, Fudan University, 2 Wulumuqi Road, Shanghai, 200040 China; 11 Global Brain Health Institute, Trinity College Dublin, 42A Pearse Street, Dublin, D02 R123 Ireland; 12 University of Manchester, Oxford Road, Manchester, M13 9P UK; 13 National Institute for Health and Care Research, UCLH/UCL Biomedical Research Centre (Hearing Theme), 149 Tottenham Court Road, London, W1T 7DN UK

**Keywords:** Hearing, Auditory, Dementia, Alzheimer’s disease, Frontotemporal dementia, Biomarkers

## Abstract

The relationship between hearing impairment and dementia has attracted significant attention, the 2024 Lancet Commission report identifying hearing loss as the largest modifiable risk factor for dementia from mid-life. The nature of this linkage between dementia and hearing remains unclear and is likely to be complex. In principle, hearing impairment could cause (directly promote), catalyze (amplify) or be a consequence of neurodegenerative pathology and cognitive decline. Here we use this framework to examine different lines of evidence for the association between hearing impairment and dementia, and consider how this evidence speaks to potential mechanisms and treatment implications. We conclude by considering practical clinical implications for management of patients with hearing impairment and dementia, the potential role for central hearing tests as ‘auditory biomarkers’ of dementia, and the need for further collaborative and mechanistically motivated research in this area.

## Introduction

**Fig. 1:**
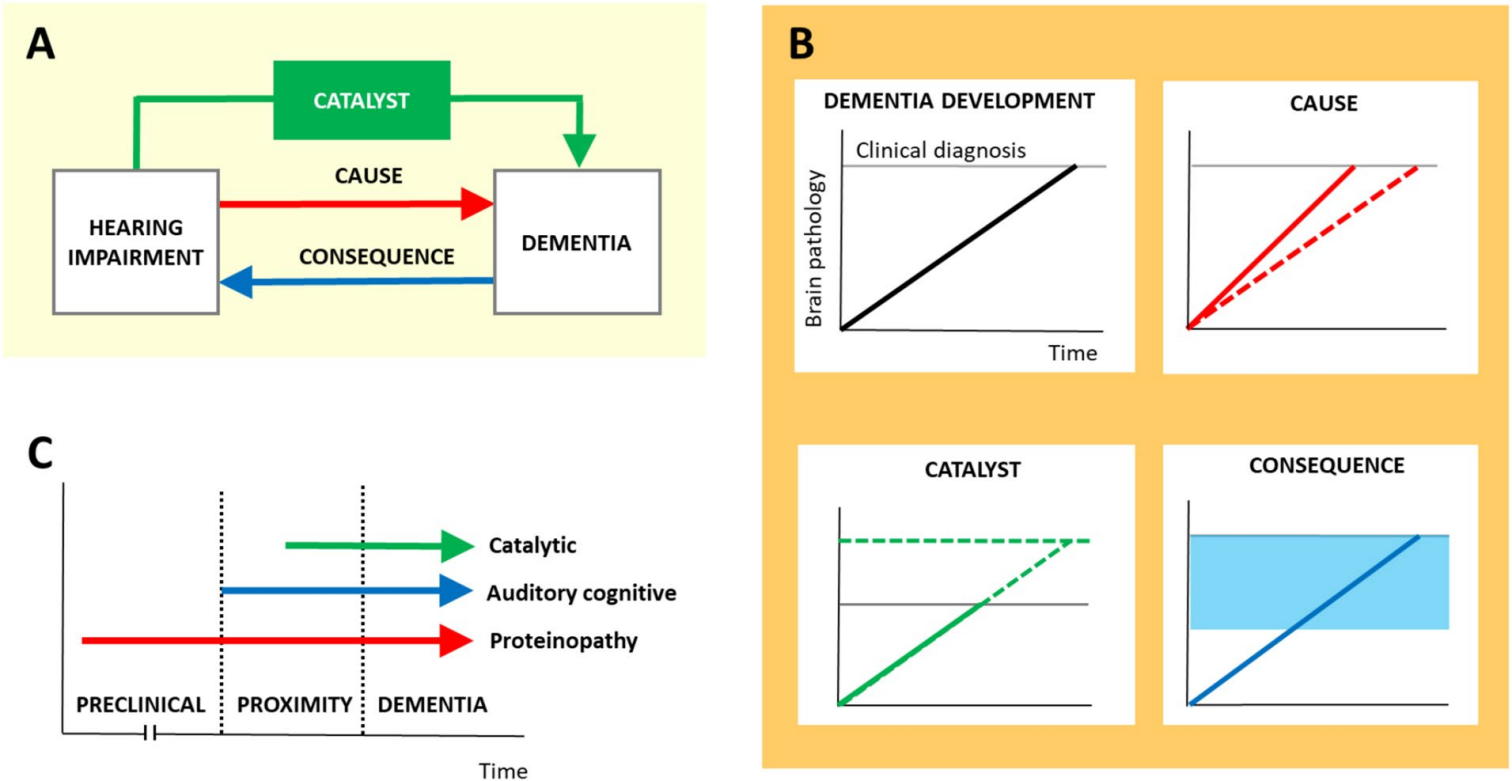
Summary of candidate mechanisms by which hearing impairment might be linked to cognitive decline in dementia. **A** Schematic showing the directionality of candidate effects – hearing loss might directly promote neurodegenerative brain pathology (**CAUSE**), result from neurodegenerative pathology (**CONSEQUENCE**) or promote other factors (e.g., impaired communication, reduced cognitive reserve) that themselves exacerbate cognitive decline and thereby amplify the effects of underlying neurodegenerative pathology (**CATALYST**). The cause and catalysis mechanisms relate to peripheral hearing impairment and the consequence mechanism to central hearing (in this context, auditory cognitive) impairment, though these can be difficult to distinguish clinically (see text). **B** Panels schematising how these candidate mechanisms might affect the detection of clinical cognitive decline, and the potential impact of hearing aids. Over time (x axis), evolution of neurodegenerative brain pathology (y axis) eventually attains a threshold level (horizontal gray line) for the emergence of cognitive dysfunction sufficient for a clinical diagnosis of dementia (**DEMENTIA DEVELOPMENT**, panel 1). If hearing impairment accelerates neurodegenerative brain pathology (**CAUSE**, panel 2) this will also tend to accelerate the emergence of clinical dementia ( compare red solid oblique line [hearing loss] in panel 2 vs black solid oblique line [no hearing loss] in panel 1). If hearing impairment amplifies the clinical effects of underlying neurodegenerative pathology (**CATALYST**, panel 3) this will tend to make clinical dementia (as diagnosed using currently standard cognitive tests) detectable at a lower level of underlying neurodegenerative brain pathology (compare the horizontal grey lines in panel 3 [hearing loss] and panel 1 [no hearing loss]) . If hearing impairment is an early result of underlying neurodegenerative brain pathology (**CONSEQUENCE**, panel 4) this may be detectable using tests that assess auditory cognition before cognitive decline becomes evident on standard cognitive tests (panel 4, shaded blue area). Dashed lines (panels 2 and 3) indicate the potential impact of hearing aids on the neurodegenerative process and/or emergence of clinical dementia in each scenario; standard amplification-based hearing aids are not anticipated to substantially benefit auditory brain dysfunction (panel 4). **C** These mechanisms are not mutually exclusive and their effects are likely to interact in the individual patient, their relative importance varying over time and with proximity to clinical dementia onset (time courses of effects coded here as overlapping arrows). Midlife hearing loss promoting pathogenic protein deposition and spread would alter the overall preclinical trajectory of neurodegenerative disease (a process potentially extending over decades, and indicated here schematically rather than to scale; effects on proteinopathy, red arrow). Auditory brain dysfunction resulting from evolving neurodegenerative pathology would produce deficits on tests of auditory cognition, potentially as an early signal of proximity to clinical dementia onset as well as in established dementia (effects on auditory cognition, blue arrow); while the amplifying effects of reduced auditory input on cognitive decline would also begin to act before clinical dementia onset but advance the onset of clinical dementia (catalytic effects, green arrow). Note that the sequence of catalytic and auditory cognitive effects shown here is speculative; catalytic effects could potentially lead auditory cognitive effects. However, all three mechanisms may be operating (and interacting synergistically) around the time of dementia onset

The link between hearing impairment and dementia has emerged as a key issue in the health of older people, at the interface of neurology, audiology, psychiatry, and public health. Successive reports from the Lancet Commission on dementia prevention, intervention, and care in 2017, 2020 and 2024 have identified hearing loss as a major dementia risk factor [1], and hearing impairment has been foregrounded as a potential cause or driver of dementia, with the corollary that hearing aids may delay or prevent cognitive decline [2, 3]. However, despite widespread attention and a number of large-scale studies, the relationship between hearing impairment and dementia has not been clarified (Table 1) and is likely to be complex. In principle, hearing impairment could be linked pathophysiologically to cognitive decline and dementia in three major ways: as a cause (directly promoting neurodegenerative pathology), a catalyst (promoting other factors that exacerbate cognitive decline and amplifying the effects of incipient neurodegenerative pathology) or a consequence (reflecting the effects of neurodegenerative pathology) (Fig. 1). These mechanisms are likely to interact. A fourth possible type of association, ‘conjunction’ — shared neurodegenerative pathology jointly affecting both brain and cochlea — we do not consider further here, given that this is poorly substantiated and would not represent a true pathophysiological linkage between hearing and dementia. Nevertheless, vascular ischemic pathology does commonly affect both ear and brain, and inflammatory, mitochondrial and other conjoint effects have also been proposed [4, 5].

**Table 1:** Large longitudinal studies reporting associations between objective hearing impairment and dementia

Study	Year	Cohort description	Follow-up	Covariates	Dementia risk (Hazard ratio)
Baltimore Longitudinal Study of Aging [13] (USA)	2011	*N* = 639 (360 male; 52 Black, 580 White, 7 other), age range 36–90 58 incident dementia cases	11.9 years (median)	Age, sex, race Education, smoking Diabetes, hypertension	1.27 (1.06–1.50) per 10 dB hearing loss
Health, Aging and Body Composition Study [18] (USA)	2017	*N* = 1,889 (893 male; 625 Black, 1,264 White), mean age 75.5 229 incident dementia cases	9 years	Study site Age, sex, race Education, smoking Diabetes, hypertension, stroke	1.14 (1.03–1.26) per 10 dB hearing loss
Health, Aging and Body Composition Study [16] (USA)	2019	*N* = 1,810 (872 male; 630 Black), mean age 77.4 336 incident dementia cases	10 years	Age, sex, race Education, alcohol use, exercise, smoking Cardio/cerebrovascular disease, diabetes, hypertension	1.25 (1.01–1.55)
Health, Aging and Body Composition Study [17] (USA)	2022	*N* = 2,061 (989 male; 776 Black), mean age 74.0 223 incident dementia cases^b^	10 years	Study site Age, sex, race Education, living alone, marital status BMI, diabetes, hypertension, stroke	1·99 (1·47–2·68)
Mayo Clinic Study of Aging [14] (USA)	2022	*N* = 1,200 (593 male; 1,193 non-Hispanic or Latino, 1,177 White), mean age 76 207 incident dementia cases	7 years (mean)	Age, sex Education, smoking ApoE4, diabetes, hypertension Hearing rehabilitation (hearing aid / cochlear implant)	0.99 (0.89–1.12) per 10 dB hearing loss
Hearing Examinations in Southern Denmark Database [65] (Denmark)	2024	*N*= 573,088 (48% male), mean age 60.8 23,023 incident dementia cases	8.6 years (mean)	Calendar year Age, sex, country of origin Cohabiting status, education, income, occupation, other socioeconomic variables Cardiometabolic diseases (diabetes, stroke, IHD, heart failure)	All dementia: 1.07 (1.04–1.11) AD: 1.11 (1.05–1.18)
Atherosclerosis Risk in Communities Neurocognitive Study [21] (USA)	2025	*N* = 2,946^c^ (1,195 male; 637 Black), mean age 74.9 239 incident dementia cases	6.5 years (median)	Age, sex, race Education, smoking ApoE4, BMI, diabetes, hypertension, stroke	1.67 (1.18–2.38)

The Table summarizes published studies meeting criteria for inclusion in the 2024 Lancet Commission report on dementia prevention, intervention and care [1], as follows: a cohort of at least 500 cognitively healthy people followed for at least 5 years; included incident dementia as an outcome; adjusted for age and cardiovascular risk factors; used pure-tone audiometry to measure hearing; included a hazard ratio estimate. Restricting the presentation to these rigorous, high quality studies omits a large number of other studies investigating the association of hearing impairment and dementia (see text); the table also excludes one study included in the 2024 Lancet Commission report, as hearing loss in the follow-up cohort was assessed using a whisper test, rather than pure-tone audiometry [15]. **a** likely participant overlap between these studies, which draw from the same wider cohort [16–18]; **b** hearing loss with and without depressive symptoms groups were combined; **c** sub-cohort who had pure-tone audiometry; *AD* Alzheimer’s disease, *ApoE4* apolipoprotein E4 allele genetic status, *BMI* body mass index, *IHD* ischaemic heart diseas

Currently, both ‘hearing impairment’ and ‘dementia’ are under-specified in published studies. Hearing impairment is often based on subjective self-reported hearing difficulties, rather than objective measurements, such as pure-tone audiometry (PTA). On the other hand, PTA correlates only loosely with daily-life hearing function [6]. Moreover, it is principally designed to detect peripheral hearing loss due to age-related or other cochlear and middle ear pathology. It does not index those real-world hearing functions — such as auditory scene navigation and perception of degraded speech [7, 8] — that depend on auditory cognitive processes. Indeed, such processes are poorly characterized using standard hearing tests. This raises the problematic distinction between so-called ‘peripheral’ and ‘central’ hearing. While certain hearing functions can in principle be characterized as peripheral or central based on the location of the structures that critically support those functions within the auditory system, attributing hearing symptoms or deficits to particular structures (e.g., the cochlea, subcortical auditory pathways or auditory cortex) is challenging, and in practice this is often a false dichotomy [8]. While particular hearing symptoms can suggest a predominantly peripheral or central pathology, few are specifically localizable, and this is compounded by extensive reciprocal interactions between different levels of the auditory system. PTA performance is affected by central cognitive processes such as attention and executive function [9, 10]. Further, pathologies of cochlea, subcortical auditory pathways and auditory cortex frequently coexist, and aging itself affects processing throughout the auditory system. Finally, standardized tests of central hearing function are presently limited [8].

Although ‘dementia’ is often used or interpreted as a diagnostic label, it is a syndromic description of acquired cognitive decline caused by a variety of diseases. The key issue from an epidemiologic perspective is to establish the link with Alzheimer’s disease (AD), which is our focus here; this requires biomarker confirmation that is often lacking in large-scale studies. A decline in cognitive performance may reflect hearing impairment per se (especially where tests rely on accurate speech perception [11]), rather than underlying neurodegenerative brain pathology. Equally, a finding of progressive brain atrophy or positive protein biomarkers does not equate to a clinical syndromic diagnosis of dementia. A related difficulty is that AD pathology often coexists with vascular ischemic changes: the latter may modify cognitive and/or hearing phenotypes, yet are challenging to quantify.

Here, acknowledging these caveats, we critically review the evidence for an association between hearing impairment and dementia risk, before distilling evidence for each of the potential mechanisms and how they might interact (Fig. 1). We highlight common issues in published studies, and outline opportunities to address these (Table 2). We go on to review the practical clinical implications posed by this synthesis and conclude by outlining a roadmap for future progress.

**Table 2:** Some issues in published studies of hearing impairment and dementia

Issue	Implications	Proposed solution
No index of real-world hearing	PTA alone is not a good predictor of real-world hearing ability and does not correlate with self-reported hearing disability measures [6]	Administer hearing symptom questionnaires with hearing tests
No objective hearing measure	Subjective hearing complaints may reflect peripheral or central hearing impairment and may be influenced by factors other than hearing impairment per se	Administer both objective peripheral and central hearing tests
Conflation of cognitive decline and dementia	A diagnosis of dementia requires progressive cognitive decline sufficient to affect daily-life functioning; cognitive test performance may be impacted by hearing loss independently of underlying dementia	Standardized cognitive assessment and usage of international diagnostic criteria for dementia diseases
Conflation of brain atrophy and dementia	Brain atrophy (especially temporal lobe atrophy involving auditory areas) may result from sensory deafferentation rather than primary neurodegeneration; dementia is a clinical syndrome	Correlative standardized dementia diagnosis
Specificity of dementia diagnosis	Dementia has many causes and is not equivalent to AD; diagnosis of AD should ideally be based on disease-specific biomarkers	Apply specific consensus diagnostic criteria; measure AD biomarkers
Short study duration	Common dementias such as AD have a presymptomatic phase extending over many years; combining different age cohorts does not reliably equate to individual longitudinal effects	Longitudinal studies with extended follow-up periods and earlier pre-symptomatic start points
Interpretation of hearing aid benefit	Prescription and compliance with hearing aids may be influenced by factors apart from hearing loss; estimated hearing aid benefit should be based on daily-life function measures as well as test scores	Randomized controlled trials of hearing interventions with daily-life functional as well as cognitive outcomes
Lack of standard central hearing measures	Studies may be blind to the possibility of reverse causation or conjoint impacts from peripheral and central hearing deficits on daily-life hearing and dementia risk	Administer central hearing (e.g., dichotic listening) tests with PTA

The Table summarizes some common issues arising when interpreting the current evidence base on the linkage between hearing impairment and dementia, with suggestions for how these issues might be addressed in future studies. *AD* Alzheimer’s disease, *PTA* pure-tone audiometry

## What is the evidence that hearing impairment is associated with dementia?

The evidence linking hearing impairment and dementia has been derived chiefly from large longitudinal cohort studies assessing the risk of incident (all-cause) dementia associated with baseline peripheral hearing loss from late middle life (Table 1). A comprehensive meta-analysis of 50 cohort studies linked hearing impairment (indexed either objectively or by self-report) to increased risk of AD, mild cognitive impairment and undifferentiated cognitive decline but not vascular dementia [12]. In the 2024 Lancet Commission report, a meta-analysis of six such studies [13–18] (each based on at least 500 participants followed for at least five years) estimated a 37% increased risk of incident dementia attributable to hearing loss, as measured using PTA and after adjusting for potentially confounding variables [1]. However, there is significant variability within the source studies [14], even among those based on overlapping cohorts [16–18] and after adjusting for confounders (Table 1). The study with the longest follow-up period to date (over 20 years) found a more modest level of risk attributable to midlife hearing loss [19]. The association between hearing impairment and dementia is likely to transcend linguistic and cultural boundaries, and has been found to be comparably strong among English and tonal [Chinese] language speakers [20].

Interpretation of the extant literature is challenging for a number of reasons (summarized in Table 2). Estimating hearing impairment based on self- or informant-led subjective reports has been found to be variably associated with apparently increased or unchanged dementia risk [20, 21]. While PTA alone may not accurately reflect real-world hearing abilities, lack of reporting (or awareness) of hearing difficulties might be a signal of emerging dementia. In addition, subjective hearing loss may be nonspecifically associated with cognitive decline but not AD biomarkers [22]. Furthermore, although incident dementia cases have sometimes been categorized by cognitive test scores [16–18], cognitive decline per se should not be equated with dementia, which implies a threshold of cognitive impairment sufficient to impact daily-life functioning. While cognitive decline often presages development of dementia, establishing conversion rates will ultimately require extended periods of longitudinal follow-up, tracking cognitive function in individuals over time. Some studies have combined groups with different comorbidities (such as depression) [17].

## How could hearing impairment be associated with dementia?

### Does hearing impairment cause dementia?

Much current clinical interest centers on the possibility that hearing impairment might cause dementia — opening up an important potentially preventative intervention in hearing aid use [3, 23]. However, the evidence for this remains relatively limited. If hearing impairment promotes the spread of neurodegenerative proteinopathies, this could, in principle, be detected using neuroanatomical or molecular pathologic markers (Fig. 1); however, neither of these modalities has clearly supported a direct causal association.

#### Neuroanatomical studies

A number of studies have reported associations between peripheral hearing impairment identified through PTA and whole brain and/or temporal lobe volume loss in cognitively normal individuals [24–26]. However, such neuroanatomical changes might arise from sensory deafferentation without requiring associated pathogenic protein deposition, and might also interact with pleiotropic genetically determined neuroanatomical phenotypes linked to sex or other factors [27]. Volume loss that is due to accelerated neurodegenerative pathology should be colocalised with disease-related atrophy profiles in patients developing clinical dementia; however, this has not been established. One recent study of patients with syndromes of AD and primary progressive aphasia found no association between PTA performance and regional gray-matter volumes [28].

#### Molecular pathologic studies

If hearing impairment accelerates the evolution of neurodegenerative proteinopathies, markers of hearing impairment might be expected to correlate with markers of neurodegenerative protein deposition even prior to the development of clinical dementia. In the case of AD, there is some evidence that PTA performance may be associated with biomarkers amyloid-beta and phosphorylated tau on brain PET [29] in cognitively healthy older adults, while subjectively reported hearing impairment was found to correlate with total CSF tau and phosphorylated tau levels in the Alzheimer’s Disease Neuroimaging Initiative (ADNI) and Chinese Alzheimer’s Biomarker and Lifestyle (CABLE) multi-centre cohort studies [26]. Other studies, however, have failed to identify an association between hearing impairment (assessed subjectively or with PTA) and AD biomarkers in cognitively healthy [30–32], cognitively impaired [33] older adults, or those with established AD relative to cognitively healthy older adults [34].

A diagnosis of neurodegenerative disease rests ultimately on the histopathological examination of brain tissue. Correlative post mortem studies of hearing impairment in AD have been based on subjectively reported hearing status rather than PTA, with mixed findings. Whereas hearing difficulty reported by cognitively healthy participants in the National Alzheimer’s Coordinating Center (NACC) database was associated with neurofibrillary tangle pathology (Braak stage), in those with clinical dementia there was no association with AD pathology (and indeed, an inverse association with amyloid plaque density) [35]. Other studies have found no association [36].

While post-mortem associations between subjective hearing impairment and AD pathology are not clearly defined, some studies have reported an association of subjective or audiometric hearing impairment with Lewy body pathology, at post mortem or on dopaminergic transporter neuroimaging [34–36]. Cerebrovascular pathology has also been variably associated with hearing impairment [36].

#### Genetic associations

Mendelian randomisation techniques have been applied to assess the role of peripheral hearing impairment as a risk factor for dementia. Overall, the evidence for a direct causal relationship between hearing impairment and dementia is not conclusive. In a two-sample and multivariable Mendelian randomisation study pooling various cohort datasets, genetic determinants of hearing loss were associated with elevated risk of Lewy body dementia and frontotemporal dementia as well as AD; the association was found to be mediated by loneliness, depression and medial temporal lobe volume [37]. Other studies have suggested a complex or nuanced relationship between hearing difficulty and AD genetic risk, based on shared predisposition to inflammation independent of Apolipoprotein allele E4 (ApoE4) genetic status [5]. Evidence has also been presented for reverse causation — a causal link from AD genetic risk to hearing impairment driven by ApoE4, suggesting that hearing impairment may be an early feature of AD [38]. A large study of polygenic risk scores for modifiable risk factors and the AD phenome (a composite of clinical, neuroimaging, CSF and neuropathological features) using two-sample Mendelian randomisation found no evidence for a causal role of hearing difficulty [39]. A genome-wide association analysis of genetic liability for AD in the UK Biobank dataset suggested that most previously reported associations (including hearing impairment as assessed by hearing aid use and speech-in-noise perception) are more likely to be a consequence of prodromal disease or selection bias rather than causal factors [40].

#### Mechanistic models

A critical issue in interpreting the risk of cognitive decline attributable to hearing impairment is to determine how a causal link might operate mechanistically. In principle, the pathologic process could involve the auditory cortex jointly with the cochlea and/or retro-cochlear pathways [41]. However, for the major neurodegenerative dementias the key question is whether disrupted auditory input could promote the spread of neurodegenerative proteinopathies at the level of cortex, hippocampus, and connected neural circuitry. Griffiths and colleagues [23] have reviewed candidate mechanisms by which midlife hearing impairment could lead to development of dementia, including common pathology affecting ascending auditory pathway and cerebral cortex, depletion of cognitive reserve due to deafferentation atrophy, occupation of cognitive resources under challenging listening conditions and altered neural activity interacting with neurodegenerative pathology in medial temporal lobe circuitry. The last is particularly noteworthy here, as a biologically plausible mechanism whereby central effects of peripheral hearing loss could promote pathogenic (Alzheimer) protein spread in hippocampal circuits.

### Does hearing impairment catalyze dementia?

A number of studies have investigated whether the link between hearing impairment and dementia is not direct, but rather mediated or ‘catalyzed’ by other factors. Hearing impairment might unmask underlying, incipient neurodegenerative processes, leading to earlier cognitive decline and dementia onset (Fig. 1). The detrimental effect of hearing impairment on neuropsychological test performance is well established [11], while Griffiths and colleagues review evidence for its impact on cognitive reserve and resource allocation [23].

#### Mediation or moderation by psychosocial factors

Hearing impairment can have a dramatic impact on communication and social functioning in an individual’s daily life, and is associated with loneliness and social isolation [42]. The impact of hearing impairment on cognitive function and/or dementia risk is influenced by social isolation, loneliness, depression [43], participation in leisure activities [44] and psychological resilience [44]. However, a study of UK Biobank data found that while hearing aids improved performance on cognitive tests, this relationship was not mediated by social isolation or depression [45]. A separate study exploring the relationship between hearing impairment (indexed from speech-in-noise perception) and dementia in the UK Biobank dataset found limited evidence for mediation through depressive symptoms and social isolation [46]. Taken together, this evidence shows that the relationship between hearing impairment, cognitive function and potential psychosocial mediators is complex, probably reflecting the multidimensional (and still relatively under-specified) status of factors such as ‘social isolation’.

#### Reduced cognitive reserve and cognitive resource reallocation

An alternative hypothesis is that people with hearing impairment consume more of their cognitive reserve for listening, which limits the neural resources, such as working memory and language processing, available for other aspects of cognitive function [23]. Such cognitive ‘compensation’ might exhaust capacity in brain areas commonly associated with dementia pathologies, particularly in the context of regional ‘disuse atrophy’ due to auditory deafferentation [23]. This would reduce resilience to AD pathology, leading to cognitive decline at an earlier stage of evolution of neurodegeneration. However, this catalytic mechanism is not specific to AD or any other dementia pathology: in the UK 1946 birth cohort, peripheral hearing impairment predicted faster rates of brain atrophy in older adults, independently of AD and cerebrovascular markers [47]. These findings are consistent with previous evidence that hearing impairment impacts neuropsychological test performance, either directly (relevant particularly to speech-based tests) or indirectly by diverting available cognitive resources (potentially relevant to any cognitive test [11]). Such a mechanism might amplify or simulate the impact of neurodegeneration.

### Is hearing impairment a consequence of dementia?

#### The concept of auditory brain dysfunction

A third hypothesis to account for the link between hearing impairment and dementia is reverse causation, which holds that the brain changes accompanying dementia (and potentially also its prodromal phase) lead to central hearing impairment [8, 41, 48] (Fig. 1). ‘Central’ in this context refers to auditory cognitive impairment. Auditory cognition is often assumed to reflect generic cognitive functions (such as attention, executive function and language processing) whereas in fact it comprises specific auditory brain operations that disambiguate sounds of interest from acoustic noise, generate stable percepts based on incoming acoustic data and associate those percepts with meaning [8]. Hearing in daily life depends on such operations, and they are likely to be affected early by neurodegenerative pathologies. This is due both to the computational complexity of auditory processing, and the anatomic distribution of auditory neural networks, which closely overlap the networks targeted in AD and other dementias [8]. Auditory brain functions are not well captured by standard neuropsychological or audiological tests; new tests that can capture these functions might sensitively detect neurodegenerative pathology [49].

There is some evidence that informant-based ratings of daily-life hearing impairment outperform PTA in predicting incident dementia [14] and correlate with neurodegenerative brain pathology [26]. Taken together, these findings suggest that auditory brain dysfunction, as indexed by everyday hearing difficulty, might generate physiologic markers of early dementia or indeed ‘proximity markers’ heralding dementia onset. This interpretation could in principle be relevant to a number of studies employing subjective reports of daily-life hearing function (Table 2). On the other hand, hearing changes measured by PTA do not invariably index peripheral hearing function, but might at least in part reflect ‘top-down’ auditory efferent pathway dysfunction due to brain pathology [8, 50].

#### Dichotic listening

Several studies have reported that worse central auditory function is associated with increased risk of cognitive decline and/or dementia [48, 51, 52]. Dichotic listening tests — in which different auditory information (usually, spoken digits or sentences) presented simultaneously to either ear must be integrated via central mechanisms — are arguably the most widely used probes of central hearing in the context of dementia. Two studies have reported associations between reduced dichotic listening performance and AD biomarkers: in 50 cognitively healthy older adults, dichotic listening (but not PTA) scores predicted brain amyloid PET positivity [31], while in 87 members of the PREVENT-AD cohort, dichotic listening performance was associated with increased CSF total and p-tau levels [53].

#### Speech-in-noise perception

Perception of spoken messages in background noise depends both on peripheral and central auditory processes; interpretation of published studies that assess hearing using speech-in-noise tests (Table 2) is therefore not straightforward. Performance on a digits-in-noise test has been shown to predict PET-derived amyloid burden in ‘younger-old’ (mean age 74.4 years) but not ‘oldest-old’ (mean age 92.7 years) participants at risk of AD [54]. A version of this test is currently employed in the largest biomedical database in the world as an objective measure of hearing function (the UK Biobank Study), and analysis of this dataset has shown impaired performance on this hearing test is associated with increased risk of incident dementia [46]. Sensitivity analyses of this dataset have found a slightly stronger risk with dementia onset within the first few years of follow-up, which would be consistent with a proximity effect due to reverse causation; however, the mechanism of the association has not been established. A pitfall with studies of this kind is the long prodromal phase (potentially, over a decade) during which brain pathology might affect hearing prior to dementia onset [49], as well as possible vascular ischemic changes in subcortical auditory pathways [8].

#### Auditory cognitive phenotypes of dementias

It is becoming evident that different dementia syndromes have distinctive auditory cognitive phenotypes associated with their underlying neurodegenerative pathologies [8, 41]. The existence of such phenotypes supports the concept that auditory brain dysfunction produces clinically relevant hearing changes, and is difficult to reconcile with a purely unidirectional effect of peripheral hearing loss in promoting neurodegeneration. However, this issue is complicated by the frequent lumping together of different brain pathologies under a single ‘dementia’ umbrella in published studies.

AD is characteristically associated with impaired auditory scene analysis — the cognitive processes involved in tracking and identifying sounds of interest in noisy environments [55–57]. Deficits of auditory scene analysis have been observed both in the typical amnestic variant and the rarer visuospatial presentation of posterior cortical atrophy, while caregivers of patients with the language-led variant of AD (logopenic aphasia) report increased difficulty hearing in background noise as an early symptom of the disease [58], suggesting that such deficits are a generic signature of AD pathology. AD also impairs comprehension of acoustically degraded speech [7]. Early difficulties with dichotic listening (disambiguating superimposed sounds) and speech-in-noise perception are likely to be pathophysiologically related features of the AD auditory phenotype.

Other dementias produce a diverse range of auditory deficits. In the frontotemporal dementia spectrum, the behavioral variant is typically associated with abnormal emotional and behavioral reactions to auditory stimuli such as voices, environmental sounds and music [59]. The semantic variant of primary progressive aphasia produces associative auditory agnosia affecting recognition of environmental sounds and voices [60–62]. The nonfluent/agrammatic variant of primary progressive aphasia is characterized by a complex and variable auditory phenotype encompassing impaired pure tone detection [50] and deficits of rhythm, pitch and timbre perception [62–64]. Hearing changes also accompany Lewy body syndromes (Parkinson’s and dementia with Lewy bodies): though not yet fully defined, auditory manifestations in these diseases include auditory hallucinations (usually indistinct nonverbal sounds, ‘muffled’ voices or music) and deficits of auditory scene, spectrotemporal and rhythm perception, probably attributable to dopaminergic and cholinergic synaptic dysfunction [8, 41].

### Interacting mechanisms

These candidate mechanisms linking hearing impairment and dementia are not mutually exclusive and indeed, are likely to interact synergistically, with considerable scope for vicious cycling. Significant hearing impairment of any cause (if untreated) would tend to promote social dysfunction, degrade performance on cognitive tests and divert cognitive resources, whether or not it directly drives (or indeed, reflects) neurodegeneration. Auditory brain dysfunction due to neurodegenerative pathologies is likely to coexist with other (non-auditory) cognitive deficits and reduced cerebral resilience. Mechanisms might promote and amplify one another: neurodegeneration triggered by peripheral hearing loss might involve auditory cortical functions, while auditory cortical dysfunction may have top-down effects on cochlear function and itself impact neural compensatory mechanisms and cognitive reserve [8]. However, the relative importance of different mechanisms is likely to vary depending on proximity to dementia onset (see Fig. 1). In the years preceding and after dementia onset, all three mechanisms may be operating together, creating challenging problems of interpretation for studies with short follow-up times in older populations.

## Clinical implications

### Hearing rehabilitation

To the extent that hearing impairment is a cause or catalyst of dementia, it follows that hearing rehabilitation may help protect cognitive function. As illustrated by Fig. 1, in general addressing hearing impairment is anticipated to delay rather than entirely prevent the onset of dementia; evidence for ‘prevention’ is determined largely by the duration of study follow-up. Nevertheless, a substantial delay in dementia onset would still have huge benefits for health care systems as well as individuals. There is some evidence that hearing aids [3] and cochlear implants may reduce long-term risk of cognitive decline [2], though interpretation of this evidence base is not straightforward (Table 2). One Danish study found that the risk of dementia was higher among people with hearing loss who were not using hearing aids (HR 1.20, 95% CI, 1.13–1.27) than those who had hearing loss and were using hearing aids (HR 1.06, 95% CI, 1.01–1.10) [65]. However, a randomized controlled trial where participants aged 70–84 years with untreated hearing loss were randomly assigned hearing aids or the control intervention of health education, recently reported that hearing rehabilitation did not reduce 3-year cognitive decline in the total cohort, although there was some evidence from prespecified sensitivity analyses to suggest that the hearing intervention was effective in reducing cognitive change in a subgroup of participants at higher risk for cognitive decline [3]. The magnitude of cognitive benefit in the subgroup was modest (for example, mean mini-mental state examination score in the intervention group changed over three years from 28.1 (1.7) to 26.9 (2.8) compared with 27.9 (1.8) to 26.6 (2.7) in the control group). Other studies have reported no or conflicting evidence of impact of hearing aids on cognitive performance [66, 67]. Assessment of hearing benefit should include daily life functional measures as well as cognitive test scores and hearing aid compliance measures.

To the extent that hearing impairment is a consequence of neurodegenerative pathology, the key issue in managing hearing impairment is to develop acoustic environmental modifications and assistive ‘smart’ technologies that can (for example) assist auditory scene analysis by enhancing speech intelligibility relative to extraneous noise. Indeed, such approaches are well motivated to improve daily-life communication in the wider aging population. However, regardless of any impact on dementia risk, peripheral hearing should always be assessed and corrected where necessary and the National Institute for Health and Care Excellence (NICE) recommends that adults with dementia or mild cognitive impairment should have a hearing assessment every two years if they have not been previously diagnosed with hearing loss [68]. Individuals with significant hearing symptoms should be supported with hearing rehabilitation and this should be tailored toward those with cognitive impairment where appropriate. Maximizing the fidelity of peripheral auditory inputs will reduce cognitive load, and hearing impairment in the individual older person is unlikely to be purely peripheral or central in nature. Compliance with hearing aids in older listeners is poor, particularly in the setting of cognitive decline [69], for reasons that are likely multifaceted [70, 71]. This of course complicates interpretation of the epidemiologic evidence: not only does it potentially vitiate the impact of the hearing intervention, but cognitive differences between older hearing aid users and non-users may not be entirely attributable to amelioration of hearing loss.

### Opportunities for early detection of dementia: auditory biomarkers

Tests of central hearing are already demonstrating promise as predictors of dementia [49]. With long-awaited disease-modifying pharmacotherapies for AD now entering clinical practice, there is an urgent need for scalable, accurate and cost-effective tools for early dementia diagnosis and rapid evaluation of treatment response. The advent of blood-based biomarkers is set to transform AD diagnosis, but actioning a blood test result that signals the presence of AD pathology will depend crucially on widely available, clinical functional readouts of that pathology - tests that can identify where an individual person is located on the illness trajectory and the proximity of cognitive decline. Hearing tests that assess auditory brain function could act as diagnostic cognitive ‘stress tests’ alongside other modalities in cognitive clinics, supported by appropriate digital platforms. Beyond diagnosis, stratifying individual patients based on their likely auditory disability will inform personalized management.

Implementation of auditory ‘dementia screening’ tests will present a major challenge for brain health clinics and clinical memory and audiology services, and will require clear best practice guidelines, robust onward referral pathways and ideally multidisciplinary clinics, to ensure patients are managed, counseled and supported appropriately. This is set to become a particularly pressing issue in the dawning era of blood-based biomarkers and disease-modifying therapies for dementia.

## Conclusions and future directions

Hearing impairment is likely to be linked to dementia via at least three, plausibly interacting mechanisms, each raising challenges and opportunities for future research and clinical practice.

Clearer operational definitions of hearing impairment and dementia, following international diagnostic criteria, are needed to shape the research agenda — these should ideally be supported by quantitative metrics and biomarkers, while acknowledging that daily-life hearing dysfunction is not well captured on PTA and dementia is not synonymous with AD. Clarifying the role of peripheral hearing loss in promoting or modulating neurodegenerative pathology will require large-scale longitudinal studies and ultimately, biomarker and histopathological confirmation, supported by biologically plausible mechanistic models [23]; longer follow-up periods (ideally over 10 years) will be particularly important to properly model all candidate mechanisms, including both direct and reverse causation effects. Incorporation of innovative modalities in future work — such as Mendelian randomisation, functional neuroimaging and electrophysiology — promises to help elucidate the pathophysiological mechanisms linking hearing impairment and dementia.

New, standardized, widely translatable tests of auditory brain function relevant to target neurodegenerative pathologies (for example, clinical tests of auditory scene analysis for AD) are needed — to allow peripheral and central hearing functions to be evaluated in tandem, and also to capitalize on their potential as auditory physiologic biomarkers, ‘stress tests’ and proximity signals of imminent dementia onset. This in turn could open up a new era in managing hearing impairment in older people at risk of dementia or with clinically evident dementia. More work is needed both to establish the benefit of hearing aids and to develop new, dementia-friendly ‘smart’ hearing technologies, environmental ‘soundscaping’ and rehabilitative interventions such as auditory cognitive training.

Seizing the opportunities presented by this complex landscape will entail a paradigm shift among audiological and dementia researchers and clinicians — a greater appreciation of the complexity of the linkage between hearing impairment and dementia including the potential for reverse causation, and above all, interdisciplinary education and collaboration. This is a key clinical issue for aging populations globally, and the reach of future progress will depend on broadening the current research base to embrace socio-culturally and linguistically diverse, aging populations [20]. As a universal sensory function, hearing is ideally placed to transcend linguistic, educational and cultural barriers.
