# Correction: Hearing impairment and dementia: cause, catalyst or consequence?

**DOI:** 10.1007/s00415-025-13421-5

**Published:** 2025-10-21

**Authors:** Benjamin A. Levett, Avinash Chandra, Jessica Jiang, Nehzat Koohi, Dale Sharrad, Lucy B. Core, Jeremy C. S. Johnson, Madison Tutton, Tim Green, Dona M. P. Jayakody, Jin-Tai Yu, Iracema Leroi, Charles R. Marshall, Doris-Eva Bamiou, Chris J. D. Hardy, Jason D. Warren

**Affiliations:** 1 Dementia Research Centre, UCL Institute of Neurology, Queen Square, UCL, 8-11 Queen Square, London, WC1N 3AR UK; 2 Centre for Preventive Neurology, Wolfson Institute of Population Health, Queen Mary University of London, Charterhouse Square, London, EC1M 6BQ UK; 3 Department of Clinical and Movement Neurosciences, Institute of Neurology, UCL, 33 Queen Square, London, WC1N 3BG UK; 4 The Ear Institute, UCL, 332 Grays Inn Rd, London, WC1X 8EE UK; 5 National Hospital for Neurology and Neurosurgery, Queen Square, London, WC1N 3BG UK; 6 Basic and Clinical Neuroscience, King’s College London, 5 Cutcombe Road, London, SE5 9RT UK; 7 Eargym Ltd, 63-66 Hatton Garden, London, EC1N 8LE UK; 8 Speech, Hearing and Phonetic Sciences, UCL, Chandler House, 2 Wakefield Street, London, WC1N 1PF UK; 9 University of Western Australia, 35 Stirling Hwy, Crawley, WA 6009 Australia; 10 Huashan Hospital, Fudan University, 2 Wulumuqi Road, Shanghai, 200040 China; 11 Global Brain Health Institute, Trinity College Dublin, 42A Pearse Street, Dublin, D02 R123 Ireland; 12 University of Manchester, Oxford Road, Manchester, M13 9P UK; 13 National Institute for Health and Care Research, UCLH/UCL Biomedical Research Centre (Hearing Theme), 149 Tottenham Court Road, London, W1T 7DN UK

**Correction: Journal of Neurology (2025) 272:402** 10.1007/s00415-025-13140-x

In the original version of this article, in Table 1, the three superscript ‘a’ markings in the ‘Study’ column was missing, so the table legend currently references a non-existent superscript ‘a’ and the last word of the table legend, ‘disease’ was spelt incorrectly as ‘diseas’.

The correct Table 1 should have appeared as shown below.

**Table 1:** Large longitudinal studies reporting associations between objective hearing impairment and dementia

Study	Year	Cohort description	Follow-up	Covariates	Dementia risk (Hazard ratio)
Baltimore Longitudinal Study of Aging [13] (USA)	2011	*N* = 639 (360 male; 52 Black, 580 White, 7 other), age range 36–90 58 incident dementia cases	11.9 years (median)	Age, sex, race Education, smoking Diabetes, hypertension	1.27 (1.06–1.50) per 10 dB hearing loss
Health, Aging and Body Composition Study [18] (USA)^a^	2017	*N* = 1,889 (893 male; 625 Black, 1,264 White), mean age 75.5 229 incident dementia cases	9 years	Study site Age, sex, race Education, smoking Diabetes, hypertension, stroke	1.14 (1.03–1.26) per 10 dB hearing loss
Health, Aging and Body Composition Study [16] (USA)^a^	2019	*N* = 1,810 (872 male; 630 Black), mean age 77.4 336 incident dementia cases	10 years	Age, sex, race Education, alcohol use, exercise, smoking Cardio/cerebrovascular disease, diabetes, hypertension	1.25 (1.01–1.55)
Health, Aging and Body Composition Study [17] (USA)^a^	2022	*N* = 2,061 (989 male; 776 Black), mean age 74.0 223 incident dementia cases^b^	10 years	Study site Age, sex, race Education, living alone, marital status BMI, diabetes, hypertension, stroke	1·99 (1·47–2·68)
Mayo Clinic Study of Aging [14] (USA)	2022	*N* = 1,200 (593 male; 1,193 non-Hispanic or Latino, 1,177 White), mean age 76 207 incident dementia cases	7 years (mean)	Age, sex Education, smoking ApoE4, diabetes, hypertension Hearing rehabilitation (hearing aid / cochlear implant)	0.99 (0.89–1.12) per 10 dB hearing loss
Hearing Examinations in Southern Denmark Database [65] (Denmark)	2024	*N*= 573,088 (48% male), mean age 60.8 23,023 incident dementia cases	8.6 years (mean)	Calendar year Age, sex, country of origin Cohabiting status, education, income, occupation, other socioeconomic variables Cardiometabolic diseases (diabetes, stroke, IHD, heart failure)	All dementia: 1.07 (1.04–1.11) AD: 1.11 (1.05–1.18)
Atherosclerosis Risk in Communities Neurocognitive Study [21] (USA)	2025	*N* = 2,946^c^ (1,195 male; 637 Black), mean age 74.9 239 incident dementia cases	6.5 years (median)	Age, sex, race Education, smoking ApoE4, BMI, diabetes, hypertension, stroke	1.67 (1.18–2.38)

The Table summarizes published studies meeting criteria for inclusion in the 2024 Lancet Commission report on dementia prevention, intervention and care [1], as follows: a cohort of at least 500 cognitively healthy people followed for at least 5 years; included incident dementia as an outcome; adjusted for age and cardiovascular risk factors; used pure-tone audiometry to measure hearing; included a hazard ratio estimate. Restricting the presentation to these rigorous, high quality studies omits a large number of other studies investigating the association of hearing impairment and dementia (see text); the table also excludes one study included in the 2024 Lancet Commission report, as hearing loss in the follow-up cohort was assessed using a whisper test, rather than pure-tone audiometry [15]. **a** likely participant overlap between these studies, which draw from the same wider cohort [16–18]; **b** hearing loss with and without depressive symptoms groups were combined; **c** sub-cohort who had pure-tone audiometry; *AD* Alzheimer’s disease, *ApoE4* apolipoprotein E4 allele genetic status, *BMI* body mass index, *IHD* ischaemic heart disease

